# Predicting Mental Health Risk from Early-Life Adversity: A Population-Based Study of Canadian Adults: Prédiction du risque pour la santé mentale liée à l’adversité en début de vie : Étude fondée sur une population d’adultes canadiens

**DOI:** 10.1177/07067437261442418

**Published:** 2026-04-20

**Authors:** Dylan Johnson, Victoria Parker, Mark Wade

**Affiliations:** 1 Department of Psychiatry and Behavioural Neurosciences, 3710 McMaster University, Hamilton, Ontario Canada; 2 Department of Applied Psychology and Human Development, 7938 University of Toronto, Toronto, Ontario Canada

**Keywords:** early-life adversity, childhood adversity, screening, common mental health disorders

## Abstract

**Objectives:**

Building on prior population-level studies, this replication study explored the predictive accuracy of retrospectively-reported early-life adversity (ELA) for individual psychopathology risk in a Canadian population survey, with measurements focused on direct/severe ELA and occurring during the COVID-19 pandemic.

**Methods:**

Nationally-representative, cross-sectional data from 7,608 Canadians surveyed in 2022 were analysed. Group-level differences were assessed via logistic regression, and predictive accuracy of ELA was tested via area under the curve (AUC) analyses.

**Results:**

Group-based analyses found that the odds of mental health problems rose with increasing ELA, albeit nonlinearly. Across psychopathology domains, predictive accuracy was poor (AUC = 0.62–0.67). Using a high-risk cut-off of ≥4 ELAs, sensitivity values were low (0.14–0.23), while specificity was high (0.93–0.94). Similarly, positive predictive values were low (0.08–0.22), while negative predictive values were high (0.92–0.97).

**Conclusions:**

ELA screening performs poorly at the individual level. While high-risk cut-offs may rule out poor mental health for individuals with fewer ELAs, it fails to accurately identify those with psychopathology. Predictive accuracy does not improve under conditions of collective stress or by focusing on direct/severe ELA. Presently, ELA screening is unsuitable for guiding intervention allocation. Further research is needed to determine whether screening can be refined to improve mental health risk prediction.

## Plain Language Summary Title

Early-life adversity and adult mental health: Is Canadian screening getting it right?

**Plain Language Summary:**

The prevention and treatment of common mental health conditions is a priority for Canadian health care providers. As experiences of childhood adversity are linked to population-level mental health problems in adults, screening individuals for these early experiences has become increasingly common. Specifically, many providers are now encouraged to incorporate questions related to early-life adversity into clinical care to guide treatment decisions. Yet, existing studies in the USA, UK, and New Zealand suggest that screening does a poor job of identifying individuals who are struggling with mental health issues. Despite this evidence, early-life adversity screening continues to be implemented in many settings, including in Canada. The current study explored if experiences of early-life adversity can accurately distinguish between Canadian adults with and without mental health problems. It is the first study to test this question in a population-level Canadian study.

Researchers analysed data from a large, representative sample of Canadian adults during COVID-19, a time of heightened stress. They looked at whether reports of past early-life adversity (i.e., direct violence/abuse) could predict who met criteria for mental health problems such as anxiety or depression. Results indicate that early-life adversity was not a reliable way to identify individuals with mental health problems. Even when focusing on direct/severe types of adversity during a period of collective stress, screening did not clearly discriminate between those with and without mental health challenges.

These findings suggest that early-life adversity screening is not an effective clinical tool to inform treatment allocation. This strategy risks missing those who need intervention and providing treatment to those who do not need it. More research is required to develop better screening procedures that include a wider range of risk and protective factors in order to guide appropriate care.

## Résumé

**Objectifs:**

S’appuyant sur des études populationnelles antérieures, cette étude de réplication a exploré la précision prédictive de l’adversité en début de vie déclarée rétrospectivement en regard du risque individuel de psychopathologie dans le cadre d’une enquête populationnelle canadienne, la mesure ayant principalement porté sur l’adversité en début de vie directe/grave survenue durant la pandémie de COVID-19.

**Méthodes:**

Les données transversales représentatives à l’échelle nationale provenant de 7 608 Canadiens interrogés en 2022 ont été analysées. Les différences au niveau du groupe ont été évaluées au moyen d’une régression logistique, et la précision prédictive de l’adversité en début de vie a été soumise à des analyses de l’aire sous la courbe (ASC).

**Résultats:**

Des analyses au niveau de groupes ont révélé que la probabilité de problèmes de santé mentale augmentait proportionnellement à l’importance de l’adversité en début de vie, bien que de manière non linéaire. Dans tous les domaines de psychopathologie, la précision prédictive était faible (ASC = 0,62–0,67). En utilisant un seuil de risque élevé supérieur ou égal à 4 événements d’adversité en début de vie, les valeurs de sensibilité étaient faibles (0,14–0,23), tandis que celles de la spécificité étaient élevées (0,93–0,94). De même, les valeurs prédictives positives étaient faibles (0,08–0,22), alors que les valeurs prédictives négatives étaient élevées (0,92–0,97).

**Conclusions:**

Le dépistage de l’adversité en début de vie est associé à de mauvais résultats au niveau individuel. Bien que les seuils de risque élevé puissent exclure un mauvais état de santé mentale chez les personnes ayant subi moins d’événements d’adversité en début de vie, ceux-ci ne permettent pas d’identifier avec précision les personnes atteintes de psychopathologie. La précision prédictive ne s’améliore pas en fonction de conditions de stress collectif ou en se concentrant sur l’adversité en début de vie directe/grave. À l’heure actuelle, le dépistage de l’adversité en début de vie ne convient pas pour orienter l’affectation des interventions. Il faudra mener d’autres projets de recherche pour déterminer si l’amélioration du dépistage permet d’améliorer la prédiction des risques pour la santé mentale.

## Introduction

The last two decades have seen a proliferation of research related to the impact of early-life adversity (ELA) on health and development.^1^ Evidence showing a greater risk of physical and mental health problems among groups with more experiences of childhood adversity (sometimes called “adverse childhood experiences” or ACEs) has served as a justification for the adoption of ELA screening frameworks across health care, policy, and educational settings.^2,3^ For instance, California allocates portions of its state budget and has legislation mandating insurance coverage for universal ELA screening, while a case study from Massachusetts advocates for school-based screening of students with the aim of intervention allocation and refinement.^4,5^ A similar pattern of use has emerged in Canada. For example, clinical guidelines from health services and medical journals in Ontario,^6,7^ British Columbia,^8^ and Alberta^9^ recommend ELA screening to inform individual treatment planning and referral services. However, the original 10-item ACEs questionnaire was designed as an epidemiological tool to identify associations between childhood adversity and population health, not for universal screening or individual clinical decision-making.^10^ Its use in clinical settings remains controversial, with limited supporting research.^11,12^ Central among the critiques of using the ACEs questionnaire, or indeed other ELA screening tools, outside of their intended purpose is a poor mapping of risk classification onto specific interventions or care pathways.^12^ An even more fundamental question in considering whether and how ELA screening should be implemented in individual care is whether scores can distinguish those who develop mental health problems from those who do not—a precondition for determining who should receive intervention.

Notably, such concerns are not unique to ELA; for example, we see similar shortcomings of screening for depression^13,14^ and suicidality^15,16^ in forecasting individual risk. This highlights a broader challenge in the mental health field—namely, that screening carries both potential benefits and risks related to accurate identification (e.g., appropriate vs. inappropriate service or medication provision) and secondary impacts such as distress arising from sensitive topics without proper training or knowing how to intervene in the moment. Such risks may be especially important to consider for ELA screening due to the nature of the questions, with qualitative data indicating concerns related to youths’ fear of upsetting their parents and parents fear of social service involvement.^17^ However, if a test can accurately predict outcomes for individuals, such precision may offset the test's risk and enhance its benefits. Unfortunately, predicting an individual's prognosis from a singular, brief tool is bound to be unreliable. This is illustrated by the example of predicting recidivism/violence risk, wherein a tool may successfully detect group-level risk, but because of overly large margins of error, is unreliable at the individual level.^18^ The implications of this phenomenon remain contested, with claims that, while flawed, such prediction models still provide quantifiable estimates that may be more accurate than a clinician's unaided, subjective judgement.^19,20^ Thus, screening tools are neither inherently useful or useless. Instead, each tool's efficacy should be appropriately and reproducibly evaluated for the context of its intended use. To this end, only a handful of studies in the USA, UK, and New Zealand have used population-level data with adequate sample sizes to directly explore the utility of ELA screening for individual risk prediction.^21–23^ Using a variant of the original ACEs questionnaire, each of these studies showed poor overall discrimination of those with and without mental health problems, regardless of whether adversity was assessed prospectively or retrospectively. However, the generalizability of these findings is unknown, and changes to policy and practice at the population level should be motivated by a robust evidence base of studies with samples that reflect the representativeness of the population.

The current replication study is the first to examine group versus individual risk prediction in a large nationally-representative Canadian sample. We note contextual and measurement differences from previous studies. First, data were collected during the COVID-19 pandemic, a period of collective stress and globally-elevated mental health problems,^24,25^ which may have increased psychopathology in our sample, particularly among those with early adversity.^26^ This putative rise in prevalence may influence test metrics, such as positive predictive values (PPVs), and perhaps even test sensitivity, as screeners are more likely to detect true positives when the target condition is more common and/or severe.^27,28^ In other words, predictive accuracy of ELA screening may be improved under conditions of increased collective stress. Second, adversity measurement in the current study centred on direct maltreatment and violence exposure experiences more strongly related to mental health risk than other experiences often included in ELA questionnaires, such as family dysfunction.^29^ Specifically, we used a variant of the short form Childhood Experiences of Violence Questionnaire (CEVQ-SF) which captures experiences specific to physical abuse, sexual abuse, and exposure to domestic violence. This may enhance the predictive accuracy of ELA screening compared to broadband measures that encompass a wider range of adversities, many of which have relatively weak associations with mental health.

## Methods

### Sample

The current study sample was drawn from a nationally-representative, cross-sectional study of Canadians conducted by Statistics Canada over the period 17th March to 31st July 2022 (Mental Health and Access to Care Survey; see Supplemental materials for further details).^30^ Participation was voluntary and data were collected under the authority of the Statistics Act governing the use of statistics in Canada under the protection of the Government of Canada. Informed consent and permission to share data for the purposes of secondary data analyses were provided by all respondents. All output was vetted by Statistics Canada. The original sample comprised 9,409 adults, with 7,983 having reported on study outcomes. An additional 375 cases were removed via listwise deletion (4.69%) for not having data on ELA, with a final study sample of *n* = 7,608. Further details regarding sample selection are presented in the Supplemental materials. The weighted sample had a mean age of 47.99 years, with 51.01% female. A completed Standards for Reporting of Diagnostic Accuracy Studies (STARD) checklist is reported in the Supplemental materials.

### Measures

*Psychiatric outcomes* were derived from the World Mental Health-Composite International Diagnostic Interview,^31^ a reliable, culturally-relevant, and structured diagnostic instrument widely used in international and epidemiological research.^32^ Outcomes in the present study included mood (i.e., major depression and bipolar depression), anxiety (i.e., generalized anxiety and social anxiety disorder), and substance use (i.e., any substance) disorders, as well suicidality (i.e., any suicidal thoughts, plans, or attempts). Questions were asked via computer-assisted interview, with items corresponding to DSM-IV diagnostic criteria (e.g., “did you ever have a period of being sad or discouraged that lasted for most of the day, nearly everyday, for 2 weeks or longer?”). All psychiatric outcomes were treated as binary variables reflecting the presence (1) or absence (0) of a disorder in the previous 12-month period. Additionally, a collapsed binary indicator of “any psychiatric outcome” was produced and coded as 1 if respondents met criteria for at least one of the above conditions and 0 otherwise.

*ELA* was measured using an adapted implementation of the CEVQ-SF^33^ in the Mental Health and Access to Care Survey (MHACS) Childhood Experiences (CEX) module. Specifically, this measure assesses: (1) witnessing a parent or other adult in the household being hit by another adult; (2) being slapped on the face, head, or ears, or hit or spanked with a hard object; (3) being pushed, grabbed, shoved, or having an object thrown at one to cause harm; (4) being kicked, bitten, punched, choked, burned, or otherwise physically attacked; (5) being forced or pressured into unwanted sexual activity through threats, physical restraint, or harm; and (6) experiencing unwanted sexual touching, including kissing, fondling, or grabbing. Each item was dichotomized to reflect the presence (1) or absence (0) of the adversity prior to age 16 years. Weighted item percentages and further ELA measurement details are reported in the Supplemental materials.

### Analysis

First, we examined group differences in the odds of a mental health diagnosis on the basis of the number of ELAs reported (i.e., 1, 2, 3, or ≥4 compared to 0) in order to assess mental health risk at the population or group level as a function of increasing ELA. Second, for individual predictive accuracy, a continuous measure of ELA was used to calculate area under the curve (AUC) values. This continuous measure of ELA represented the number of unique ELAs reported (range = 0–6). AUC values range from 0.5 (chance level) to 1.00 (perfect discrimination), with values between 0.5 and 0.6 suggesting very poor discrimination, 0.6 and 0.7 suggesting poor discrimination, 0.7 and 0.8 suggesting fair discrimination, 0.8 and 0.9 suggesting good discrimination, and 0.9 and 1.0 suggesting excellent discrimination. Finally, we quantified individual classification using a commonly employed high-risk ELA cut-off score (≥4 vs. 0–3 ELAs)^2^ to derive measures of sensitivity, specificity, and PPVs and negative predictive values (NPVs). Additionally, to compare with other studies^22^ and account for a reduced set of items relative to the ACEs questionnaire,^10^ we examined classification accuracy across all potential cut-offs: ≥1 ELAs (ref = 0 ELAs), ≥2 ELAs (ref = 0–1 ELAs), and ≥3 ELAs (ref = 0–2 ELAs). A replicated sensitivity analysis was conducted for lifetime (instead of past 12 months) psychiatric outcomes, as well as for “any psychiatric outcome”. All analyses were conducted in SAS and incorporated study survey and bootstrapped weightings from Statistics Canada to ensure a nationally-representative design. Further details are provided in the Supplemental materials.

## Results

The prevalence of 12-month mental health problems was highest for anxiety disorders (9.81%) and lowest in suicidality (3.58%) (Table 1). These rates are higher than those reported a decade earlier in the comparable 2012 Canadian Community Health Survey, except for substance use disorders, which have declined slightly over time.^34^ Overall, rates of 12-month comorbidity between disorders were low. The most common cooccurring diagnoses were mood and anxiety disorders (2.16%), followed by mood and anxiety disorders with suicidality (0.76%), and mood disorders with suicidality (0.64%). All other two- and three-way combinations each occurred in fewer than 1% of respondents.

**Table 1.** Predictive Accuracy for Past 12-Month Mental Health Problems Based on Early-Life Adversity (*n* = 7,608).

Mental Health Outcome (Past 12-Months)	Weighted Prevalence	AUC (Continuous Adversity Risk Calculation)	Dichotomous ELA Risk Classification					
			ELA Cut-off	Odds Ratio (95% CI)	Sensitivity	Specificity	PPV	NPV
Anxiety	9.81	0.62	≥1	1.85 (1.51–2.26)	0.62 (0.58–0.67)	0.53 (0.51–0.54)	0.12 (0.11–0.13)	0.93 (0.92–0.94)
			≥2	2.30 (1.88–2.82)	0.42 (0.37–0.47)	0.76 (0.75–0.77)	0.15 (0.13–0.17)	0.93 (0.92–0.94)
			≥3	2.53 (2.00–3.18)	0.28 (0.24–0.32)	0.87 (0.86–0.88)	0.18 (0.15–0.21)	0.92 (0.91–0.93)
			≥4	3.04 (2.26–4.08)	0.16 (0.13–0.20)	0.94 (0.93–0.95)	0.22 (0.17–0.26)	0.92 (0.91–0.93)
Mood	8.21	0.64	≥1	2.14 (1.72–2.65)	0.66 (0.61–0.70)	0.53 (0.51–0.54)	0.11 (0.10–0.12)	0.95 (0.94–0.95)
			≥2	3.04 (2.47–3.75)	0.49 (0.44–0.53)	0.76 (0.75–0.78)	0.15 (0.13–0.17)	0.94 (0.94–0.95)
			≥3	3.02 (2.39–3.80)	0.32 (0.27–0.36)	0.87 (0.86–0.88)	0.17 (0.15–0.20)	0.93 (0.93–0.94)
			≥4	3.48 (2.60–4.65)	0.18 (0.14–0.22)	0.94 (0.93–0.95)	0.21 (0.17–0.26)	0.93 (0.92–0.94)
SUD	3.75	0.63	≥1	2.69 (1.92–3.76)	0.71 (0.64–0.78)	0.52 (0.51–0.54)	0.06 (0.05–0.07)	0.98 (0.97–0.98)
			≥2	2.14 (1.56–2.93)	0.42 (0.34–0.49)	0.75 (0.74–0.76)	0.06 (0.05–0.08)	0.97 (0.96–0.98)
			≥3	2.00 (1.39–2.87)	0.25 (0.19–0.32)	0.86 (0.85–0.87)	0.07 (0.05–0.08)	0.97 (0.96–0.97)
			≥4	2.29 (1.47–3.55)	0.14 (0.09–0.19)	0.93 (0.93–0.94)	0.08 (0.05–0.11)	0.96 (0.96–0.97)
Suicidality	3.58	0.67	≥1	3.60 (2.50–5.19)	0.77 (0.70–0.83)	0.52 (0.51–0.54)	0.05 (0.04–0.06)	0.98 (0.98–0.99)
			≥2	4.43 (3.20–6.12)	0.59 (0.51–0.67)	0.75 (0.74–0.77)	0.08 (0.06–0.10)	0.98 (0.98–0.99)
			≥3	4.27 (3.09–5.90)	0.41 (0.33–0.48)	0.86 (0.85–0.87)	0.09 (0.07–0.12)	0.98 (0.97–0.98)
			≥4	4.27 (2.85–6.40)	0.23 (0.16–0.30)	0.94 (0.93–0.94)	0.11 (0.08–0.15)	0.97 (0.97–0.98)

*Note*. ELA = early-life adversity; CI = confidence interval; NPV = negative predictive value; PPV = positive predictive value; SUD = substance use disorder; AUC = area under the curve.

**Figure 1.**
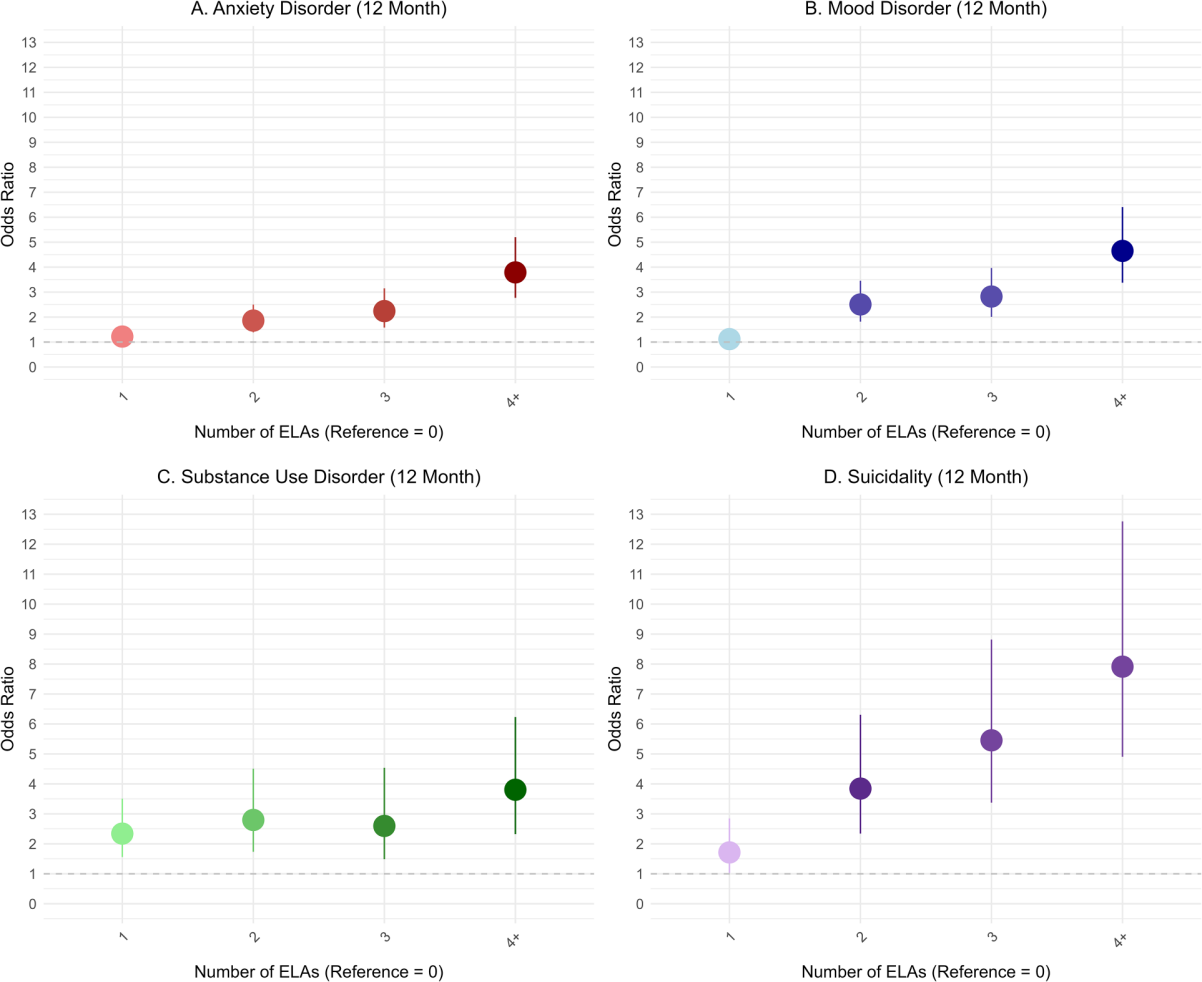
Bivariate logistic regression model results on the association between the number of ELAs and past 12-month mental health problems (*n* = 7,608). ELA prevalences were: 0 (51.2%), 1 (23.1%), 2 (11.0%), 3 (7.7%), ≥4 (7.0%). ELA = early-life adversity.

In terms of group-based analyses, as the number of ELAs increased, the odds of all mental health problems also increased, though not necessarily in a linear fashion (Figure 1). Compared to individuals with no ELAs, those with one ELA had significantly higher odds of suicidality and substance use, but not of depression or anxiety. Once two ELAs were reported, the odds of all mental health problems increased significantly by nearly twofold or greater, compared to those with no ELAs. For those reporting four or more ELAs, the odds of mental health problems were substantially higher compared to those reporting no ELA, ranging from about fourfold greater for depression, anxiety, and substance use, to nearly eightfold for suicidality.

In terms of individual risk prediction, continuous adversity score discrimination was poor for anxiety disorders (AUC = 0.62), mood disorders (AUC = 0.64), suicidality (AUC = 0.67), and substance use disorders (AUC = 0.63). Overall, this means that the likelihood that a randomly selected person with a mental health problem had a higher ELA score than a randomly selected person without a mental health problem was not much better than chance. AUC figures are presented in the Supplemental materials.

**Figure 2.**
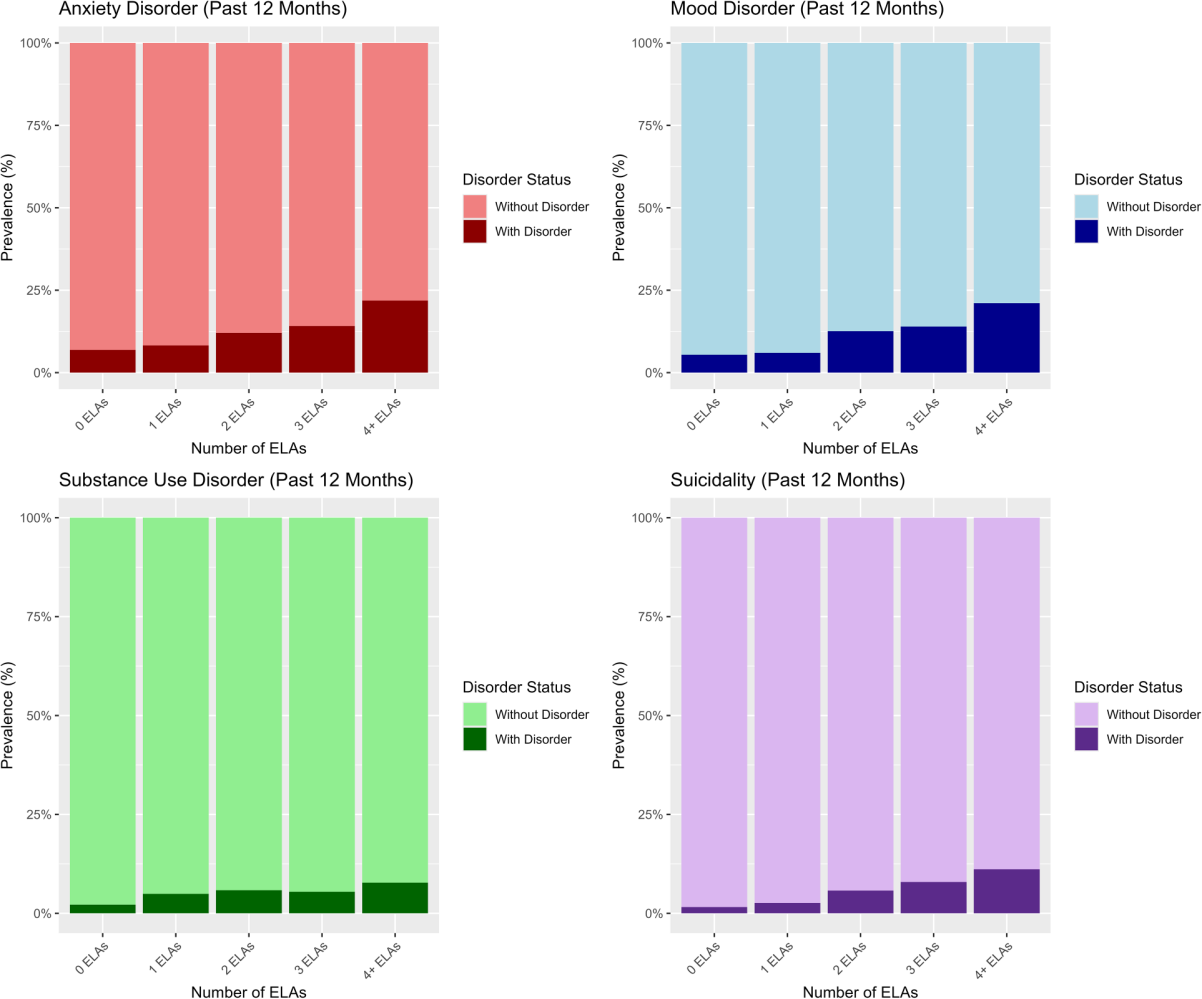
Prevalence of past 12-month mental health problems across levels of ELA. ELA prevalences were: 0 (51.2%), 1 (23.1%), 2 (11.0%), 3 (7.7%), ≥4 (7.0%). ELA = early-life adversity.

We then examined sensitivity, specificity, PPVs, and NPVs, which showed the same trend across mental health domains. Focusing on the high-risk cut-off of ≥4 ELAs, sensitivity values were very low (range = 0.14 to 0.23), suggesting the ≥4 ELA cut-off did not accurately identify most people with a mental health problem (i.e., low true positive rate). In contrast, specificity values were high across domains (range = 0.93 to 0.94), suggesting an ELA score of <4 accurately identified those without a mental health problem. PPVs were also low across domains (range = 0.08 to 0.22), suggesting that the probability that a person reporting ≥4 ELAs has a mental health problem is low. Conversely, NPVs were high across mental health domains (range = 0.92 to 0.97), suggesting that the probability that a person reporting <4 ELAs does not have a mental health problem is high. Visually, these trends can be observed in Figure 2, which presents the weighted prevalence of mental health problems across levels of ELAs. As seen in Figure 2, while the prevalence of mental health problems is proportionately higher among individuals with high levels of ELA (e.g., ≥4 ELAs) compared to those with fewer ELAs, the majority of individuals with ≥4 ELAs do not have mental health problems. Moreover, most people in the population report fewer ELAs (i.e., about half report no ELA), meaning that although, as a group, those with fewer ELAs report a lower probability of mental health problems, there are more individuals with mental health problems who report low ELAs (due to the much higher prevalence of individuals with low ELA scores in general).

We also conducted a sensitivity analysis using lifetime mental health outcomes instead of past 12-month outcomes, the results of which are presented in the Supplemental materials. Briefly, the findings mimicked those using past 12-month outcomes, suggesting the overall pattern was robust to whether recent or lifetime mental health outcomes were used. Finally, results modelling “any psychiatric outcome” for both 12-month and lifetime outcomes are presented in the Supplemental materials. Results for any psychiatric outcome were comparable to those observed for disorder-specific outcomes in terms of discrimination, dose–response relationships with ELAs, and patterns of sensitivity, specificity, PPV, and NPV.

## Discussion

The present study contributes to a growing body of literature on the utility of ELA screening, examining whether ELA scores accurately predict individual mental health risk in a nationally-representative Canadian sample. It is well established that ELA can forecast population-level risk, detecting mean differences in health outcomes between high versus low ELA groups. For example, Baldwin et al.^22^ found that each additional childhood adversity (corresponding to the 10 categories from the ACEs questionnaire) was associated with a 14% (E-Risk cohort) and 17% (Dunedin cohort) increase in relative mental health risk. Similarly, our findings show that groups of individuals reporting more ELAs have increased odds of several mental health problems, reflecting the fact that greater adversity exposure is linked to higher mental health risk at the population level.

However, mean differences in mental health risk between groups who differ on their level of ELAs do not necessarily accurately identify at-risk *individuals*. Indeed, prior research suggests that ELA screening lacks predictive discrimination, meaning it fails to reliably distinguish between those who develop mental health problems from those who do not. A substantial and growing body of evidence has considered the conceptual and methodological limitations that may underpin this poor predictive discrimination.^35–39^ Specifically, the rudimentary approach of taking a simple count of ELAs (as is done with the ACEs questionnaire and the CEVQ-SF) weighs highly heterogenous exposures as equal, and lacks the ability to capture adversity timing, intensity, or chronicity. Furthermore, serious issues have been raised regarding the content validity of ELA measurement tools, including the omission of salient early stressors such as discrimination or collective violence, as well as the failure to account for cooccurring resilience and protective factors (e.g., supportive relationships) in an individual's life that may mitigate the impact of adversity. This dilemma is further reflected in the fact that, while about half of those with ≥4 ELAs may be diagnosed with some type of mental health problem compared to about one-third of those with 0 ELAs, those with ≥4 ELAs comprise only about one-sixth of the entire population.^36^ Thus, most individuals with mental health problems will come from groups reporting fewer ELAs due to the much higher prevalence of individuals in these groups. This means that, when a particular ELA cut-off is used to predict risk (e.g., ≥4), many people may be misclassified as having a mental health problem when they do not (false positives) or not having a disorder when they do (false negatives). This same pattern was observed in the present study (Figure 2), raising questions about the utility and potential risks of ELA screening for the purpose of subsequent referral or intervention allocation.

The current study found low predictive accuracy of continuous ELA scores in discriminating those who did and did not have a past 12-month mental health problem (AUCs = 0.57–0.64). These findings are consistent with a recent reanalysis of data from the original ACEs study,^23^ as well as analyses in the E-Risk and Dunedin cohorts,^22^ all of which consistently demonstrated poor individual discrimination using either prospectively or retrospectively measured ELA. Further, Cohen and Choi^21^ found that ELA scores may produce a high number of false positives among Black male adolescents and false negatives among Hispanic female adolescents, raising equity concerns in terms of stigma, fairness, and access to care if using ELA scores as a method for mental health screening or decision-making. Combined with other critical perspectives on the potential risks associated with widespread childhood adversity screening,^37–39^ these results should cause pause among practitioners and policymakers who promote ELA screening to identify those at risk of poor health and who may require intervention.

AUC values provide an overall measure of discrimination, summarizing the relationship between sensitivity and specificity across all possible thresholds; however, they do not reveal the actual true positive rate (sensitivity) or true negative rate (specificity) of a given cut-off. Here, the results of the current study also converge with prior literature in suggesting poor individual risk classification using a high-risk cut-off of ≥4 ELAs. The findings are also similar to the results of the reanalysis of the original ACEs study^23^ in terms of both sensitivity and specificity. These findings suggest that, while most individuals without mental health problems indeed report <4 ELAs (high specificity), only a small proportion of individuals with mental health problems report ≥4 ELAs (low sensitivity). Overall, the results suggest that screening based on high-risk ELA cut-off scores may help in ruling out the likelihood of poor mental health outcomes among those reporting fewer ELAs (0–3 ELAs), but that screening does not accurately detect mental health problems among high-risk individuals (≥4 ELAs). As a precondition to intervention allocation, these results discourage the use of ELA screening as a method for determining who should receive such interventions, as doing so would likely result in unnecessary care for a large number of individuals who screen positive but do not have mental health problems (false positives). Equally concerning, as most individuals with mental health problems have fewer ELA (i.e., 0–3 ELAs), many individuals who would benefit from those interventions would not receive services based on their low-risk status. Therefore, when weighing the risks and benefits of implementing universal ELA screening, policy should consider the evidence indicating that the potential harm that such sensitive questions may impose (e.g., psychological distress, concerns about social service involvement, incorrect identification) is *not* offset by putative benefits (i.e., access to appropriate services) given its poor precision.

The current study presents a replication of the few extant population-level investigations into the utility of ELA screening,^21–23^ and is the first to generalize these findings to a nationally-representative sample of Canadian adults. We enrich the discourse by accounting for whether contextual (i.e., heightened collective stress during the pandemic) or measurement (items focused on maltreatment and abuse) differences alter the predictive accuracy of ELA screening for mental health risk. However, we note several limitations. First, the study relied on retrospective reports of ELA, which may be influenced by current mental health status and be subject to recall bias.^40,41^ Nevertheless, these factors do not fully explain the robust associations between retrospectively-reported adversity and psychopathology^42,43^ and, in some cases, do not appear to be a differentiating factor between retrospective and prospective accounts of adversity.^44^ Still, replication using a variety of retrospective and prospective measures is required to test whether differences in ELA measurement relate to differences in predictive accuracy. Second, as in past studies,^23^ low prevalence rates for certain health outcomes (e.g., suicidality) may have constrained classification accuracy, though predictive discrimination remained poor across outcomes. Relatedly, there was significant missing data on study outcomes (∼15%), which may introduce selection bias into our findings. For example, if participants with greater mental health difficulties and ELA histories were less likely to complete the survey, our results may underestimate both the prevalence of mental health problems and the strength of their association with ELA, leading to overly conservative estimates of predictive accuracy. Third, the measure of ELA used in the current study (i.e., CEVQ-SF) was originally validated primarily in adolescent samples.^28^ While it has been applied in prior adult samples,^45^ its use for retrospective reporting in adults may introduce additional measurement error. Fourth, our measure of adversity included narrower and more severe forms of ELAs (i.e., violence exposure) than those captured in the ACEs questionnaire. As these forms of adversity are associated with more significant negative outcomes, our results may be biased away from the null relative to similar cut-offs used in measures that include less severe adversity. The MHACS CEX module also does not assess emotional abuse, emotional neglect, or physical neglect, which are core components of child maltreatment in many frameworks, likely leading to an underestimation of total adversity burden. Moreover, our measure represents an adapted implementation of the CEVQ-SF embedded in the MHACS CEX module, potentially limiting direct comparability with some prior studies. Still, poor sensitivity was observed across multiple ELA cut-offs, suggesting relatively poor individual classification regardless of the threshold used. Finally, the current findings use nationally representative Canadian data, and so study implications apply to universal ELA screening of the general Canadian population. Specific use in acute treatment settings, where base rates of psychopathology are likely higher, may result in different predictive accuracy. These gaps highlight important avenues for future research to not only examine if and how predictive utility of ELA screening can be improved upon (i.e., what additional information might improve predictive accuracy), but also to continue to advance our understanding of its clinical utility.^21^

## Conclusion

ELA has gained substantial traction as a key determinant of population health. Concurrently, ELA measurement scores are being increasingly used clinically to screen individuals who may require subsequent referral or intervention, despite a lack of evidence to support this use case.^4–9^ The current results replicate previous findings suggesting that ELA does not accurately discriminate between those with and without mental health problems, and this extends to contexts characterized by high collective stress (i.e., COVID-19 pandemic) and to screeners that focus on more direct and severe adversities known to be more strongly related to mental health risk. Together, findings indicate that more research is needed to determine under what, if any, conditions ELA screening is useful in predicting individual mental health risk, and how such instruments may be improved by the inclusion of other risk and protective factors that both increase and mitigate mental health risk in the population.

## Supplemental Material

sj-docx-1-cpa-10.1177_07067437261442418 - Supplemental material for Predicting Mental Health Risk from Early-Life Adversity: A Population-Based Study of Canadian Adults: Prédiction du risque pour la santé mentale liée à l’adversité en début de vie : Étude fondée sur une population d’adultes canadiens Supplemental material, sj-docx-1-cpa-10.1177_07067437261442418 for Predicting Mental Health Risk from Early-Life Adversity: A Population-Based Study of Canadian Adults: Prédiction du risque pour la santé mentale liée à l’adversité en début de vie : Étude fondée sur une population d’adultes canadiens by Dylan Johnson, Victoria Parker and Mark Wade in The Canadian Journal of Psychiatry
