# Supplementary material for: Predicting Mental Health Risk from Early-Life Adversity: A Population-Based Study of Canadian Adults: Prédiction du risque pour la santé mentale liée à l’adversité en début de vie : Étude fondée sur une population d’adultes canadiens
Source: Can J Psychiatry. 2026 Apr 20:07067437261442418. Online ahead of print. doi: 10.1177/07067437261442418 (PMC13096011; doi:10.1177/07067437261442418)
Supplement: sj-docx-1-cpa-10.1177_07067437261442418 - Supplemental material for Predicting Mental Health Risk from Early-Life Adversity: A Population-Based Study of Canadian Adults: Prédiction du risque pour la santé mentale liée à l’adversité en début de vie : Étude fondée sur une population d’adultes canadien [file sj-docx-1-cpa-10.1177_07067437261442418.docx]

Supplemental Information

for

**Predicting mental health risk from early-life adversity: A population-based study of Canadian adults**

in

***The Canadian Journal of Psychiatry***

Dylan Johnson, PhD, Victoria Parker, M.A., Mark Wade, PhD, C.Psych

**Mental Health and Access to Care Survey (MHACS)**

**Survey Overview**The Mental Health and Access to Care Survey (MHACS) 2022 is a cross-sectional, nationally representative survey of Canadians aged 15 years and older living in the ten provinces. The survey aims to assess mental health status across illness and well-being continuums, evaluate access to and utilization of mental health services, and examine the impact of the COVID-19 pandemic on mental health outcomes. Excluded from the survey were persons living on reserves or other Aboriginal settlements, full-time members of the Canadian Forces, and those residing in collective dwellings, such as institutional residences.

**Data Collection**Data were collected between March 17 and July 31, 2022, using Internet-Based Electronic Questionnaires (EQ) administered via interviewer-led telephone interviews (iEQ). Respondents were not permitted to self-complete the survey, ensuring consistency with prior iterations of the CCHS-Mental Health. Interviewers received extensive training on survey content, and responses were entered directly into a computerized questionnaire at the time of the interview. This approach reduced transcription errors, improved data security through encryption and firewalls, and minimized processing time. Participation was voluntary, and confidentiality protections were applied according to Statistics Canada regulations.

**Sampling and Weighting**

The MHACS used a complex, single-frame survey design based on the 2021 Census of Population. The sample involved stratified selection with oversampling of specific population groups to allow disaggregated and intersectional analyses. Survey weights were calculated through a multi-step process that incorporated:

- Initial design weights based on the inverse probability of selection and Census weights
- Adjustments for ineligible/out-of-scope respondents
- Non-response adjustments using response homogeneity groups
- Winsorization to reduce extreme weight impact
- Post-stratification to align weights with population benchmarks for age, gender, and population groups

The MHACS employs a complex survey design; to account for this, Statistics Canada generated 1,000 sets of bootstrap replicate weights (BSW1–BSW1000) for variance estimation. Each set of bootstrap weights was derived from the initial design weights assigned to respondents, with both phases of the sampling design (the 2021 Census long-form sample selection and the MHACS subsample selection) incorporated through a generalized bootstrap methodology. These bootstrap weights were then adjusted in the same way as the survey weights to preserve representativeness. Because this method can yield negative weights, a transformation was applied to stabilize variability, requiring the use of rescaling factors when computing sampling errors. Specifically, variances must be multiplied by 9 and standard errors and coefficients of variation (CVs) by 3 (implemented in SAS via Fay adjustment factor). Analyses that use the bootstrap weights with this adjustment produce correct standard errors and confidence intervals, and the final weighted estimates are considered nationally representative. Full technical details of weight construction and rescaling are provided in the MHACS User Guide.

**Sample Selection**

Excluded (n=2,253)

- <18 years of age (n=452)
- Missing outcome data (n=1,426)
  - Depression (n=416)
  - Anxiety (n=554)
  - Substance Use (n=683)
  - Suicidality (n=102)
- Missing ACEs data (n=375)

♦  Other reasons (n= )

Final sample (n=7,608)

Assessed for eligibility (n=9,861)

| Table S1. Standards for Reporting of Diagnostic Accuracy Studies (STARD) Checklist | | | |
| --- | --- | --- | --- |
| **Section & Topic** | **Item** | **Description** | **Location** |
| TITLE / ABSTRACT | 1 | Identification as a diagnostic accuracy study | Pages 1–2 |
|  | 2 | Structured summary | Page 2 |
| INTRODUCTION | 3 | Scientific and clinical background, including intended use and clinical role | Pages 3–4 |
|  | 4 | Study objectives and hypotheses | Page 5 |
| METHODS |  |  |  |
| Study Design | 5 | Prospec­tive vs retrospective design | Page 5 |
| Participants | 6 | Eligibility criteria | Pages 5–6; Supplement |
|  | 7 | Basis for identifying participants | Pages 5–6; Supplement |
|  | 8 | Setting, location, and dates | Page 5 |
|  | 9 | Consecutive, random, or convenience series | Supplement |
| Test Methods | 10a | Index test description | Pages 6–7 |
|  | 10b | Reference standard description | Page 6 |
|  | 11 | Rationale for reference standard | Page 6 |
|  | 12a | Index test positivity cut-offs | Page 7 |
|  | 12b | Reference standard thresholds | Page 6 |
|  | 13a | Blinding of index test performers | Not applicable; secondary data |
|  | 13b | Blinding of reference standard assessors | Not applicable; secondary data |
| ANALYSIS | 14 | Methods for estimating diagnostic accuracy | Page 7 |
|  | 15 | Handling indeterminate results | CIDI is binary and all individuals received an outcome |
|  | 16 | Handling missing data | Page 6 |
|  | 17 | Analyses of variability | Page 7 and Supplement |
|  | 18 | Intended sample size and determination | Pages 5–6 |
| RESULTS |  |  |  |
| Participants | 19 | Flow of participants | Supplement (p. 3) |
|  | 20 | Baseline demographic and clinical characteristics | Page 6 |
|  | 21a | Distribution of disease severity | Not applicable; outcomes dichotomous |
|  | 21b | Distribution of alternative diagnoses | Not applicable; population-level classification |
|  | 22 | Time interval between index test and reference standard | Not applicable |
| TEST RESULTS | 23 | Cross tabulation of index test vs reference standard | Not reported; Statistics Canada cell-size restrictions |
|  | 24 | Diagnostic accuracy estimates with precision | Pages 8–9; Supplement |
|  | 25 | Adverse events | Not applicable |
| DISCUSSION | 26 | Study limitations | Pages 13–14 |
|  | 27 | Implications for practice | Page 15 |
| OTHER INFORMATION | 28 | Registration | Not applicable |
|  | 29 | Protocol access | Not applicable |
|  | 30 | Funding and role of funders | Not applicable |

**Childhood Experiences (CEX) Module - MHACS**

Childhood adversity was assessed using the Childhood Experiences (CEX) module of the 2022 Mental Health and Access to Care Survey administered via computer-assisted telephone (CATI) and interview (ITAO). The CEX module was administered to respondents aged 18 years and older and inquired retrospectively about experiences occurring before age 16 in the home, school, or neighbourhood.

The module comprised six core items. Respondents were asked: (1) how often they witnessed violence between adults in the home; (2) how often an adult slapped or hit them with a hard object; (3) how often an adult pushed, grabbed, shoved, or threw something at them; (4) how often an adult physically attacked them (e.g., kicked, punched, choked, or burned them); (5) how often an adult forced or attempted to force them into unwanted sexual activity through threats or physical coercion; and (6) how often an adult touched them in a sexual way against their will.

These items reflect an adapted implementation of the Childhood Experiences of Violence Questionnaire–Short Form (CEVQ-SF) within the MHACS CEX module. Compared with the original CEVQ-SF, the MHACS adaptation omits the CEVQ-SF bullying items and the standalone physical punishment (spanking) item, expands sexual abuse to two distinct items (forced sexual activity and unwanted sexual touching), and includes a dedicated item on exposure to intimate partner violence (witnessed adult violence in the home). For each item, respondents selected from standardized frequency categories: “Never,” “1 or 2 times,” “3 to 5 times,” “6 to 10 times,” or “More than 10 times,” with options for “Don’t know” and “Refusal.” In the present study, each item was dichotomized to reflect “Never experienced” endorsed (0) versus “Ever experienced” (1) as determined by endorsement of “1-2”, “3-5”, “6-10”, or “>10”. All responses were collected under assurances of confidentiality. Weighted prevalence estimates are presented in Table S1.

| Table S2. Weighted prevalence estimates of early-life adversity items | | | | | |
| --- | --- | --- | --- | --- | --- |
| **ELA Item** | **Weighted Prevalence Estimate** | | | | |
|  | **Never** | **1–2 times** | **3–5 times** | **6–10 times** | **>10 times** |
| Witnessed adult hitting another adult in the home | 85.38 | 7.62 | 2.39 | 1.17 | 3.44 |
| Slapped, hit, or spanked with something hard | 59.79 | 16.79 | 7.41 | 4.2 | 11.82 |
| Pushed, grabbed, shoved, or had something thrown | 80.48 | 9.44 | 3.45 | 1.91 | 4.72 |
| Kicked, bitten, punched, choked, burned, or physically attacked | 90.65 | 4.53 | 1.42 | 0.89 | 2.51 |
| Forced or attempted forced sexual activity | 94.11 | 3.4 | 1.03 | 0.36 | 1.1 |
| Unwanted sexual touching | 89.53 | 6.26 | 1.84 | 0.9 | 1.46 |

**Sensitivity Analyses with Lifetime Mental Health Outcomes**

Here, we replicated the primary analyses that used past 12-month outcomes, instead focusing on ***lifetime*** mental health outcomes. Prevalence rates for lifetime mental health outcomes ranged from 10.53% (suicidality) to 20.91% (anxiety disorders) (Table S1).

As with past 12-month outcomes, there was a dose-response relation between increasingly high ELAs and mental health risk in group-based analyses (Figure S1). Results largely mimicked those in the primary analysis. For lifetime mental health outcomes, the odds of elevated mental health problems were observed for those with 1 ELA compared to those with no ELAs across all mental health outcomes, whereas in the primary analyses this was only observed for suicidality and substance use. The magnitude of the odds for lifetime mental health problems was largely comparable to past 12-month problems, but was slightly larger for lifetime mental health problems at lower levels of ELAs (i.e., 1 or 2 ELAs compared to no ELAs).

In terms of individual risk prediction, continuous adversity score discrimination was very poor to poor for anxiety disorders (AUC=0.64), mood disorders (AUC=0.59), substance use disorders (AUC=0.58), and suicidality (AUC=0.61). Thus, as with past 12-month mental health problems, discrimination of those with and without mental health problems on the basis of their history of adverse childhood experiences was poor and only marginally better than chance.

For classification accuracy, patterns of sensitivity, specificity, positive predictive value (PPV) and negative predictive value (NPV) mirrored those in the primary analyses. Focusing on the high risk cut-off of ≥4 ELAs, sensitivity values were very low (range = .12 to 17, though note that this could not be estimated for the anxiety model due to Statistics Canada restrictions on data release for small samples), suggesting the ≥4 ELAs cut-off did not accurately identify most people with a mental health problem (i.e., low true positive rate). In contrast, specificity values were high across mental health domains (range = .94 to .95), suggesting an ELAs score of <4 accurately identified those without a mental health problem. While higher than for past 12-month outcomes, PPV values were still low across domains (range = .25 to .34), suggesting that the probability that a person reporting ≥4 ELAs has a mental health problem is relatively low. In contrast, NPVs were higher across mental health domains (range = .81 to .91), though lower than for past 12-month outcomes, suggesting the probability that a person reporting <4 ELAs does not have a mental health problem is relatively high.

Visually, these trends can be observed in Figure S2, which presents the weighted prevalence of mental health problems across levels of ELAs. The pattern is similar to what was observed in the primary models although, as expected, prevalence rates are overall higher given the focus on lifetime compared to past 12-month outcomes. Generally, while the group of individuals with ≥4 ELAs had a greater prevalence of mental health problems compared to those with fewer ELAs, most people with ≥4 ELAs had no problems. Moreover, given the much higher overall prevalence of individuals with lower levels of ELAs, the majority of individuals with mental health problems actually reported low levels of ELAs. Together, these results support the idea that ELAs do not discriminate well between those without and without lifetime mental health problems, similar to past-12 month problems.

| ***Table S3*.** Predictive accuracy for lifetime mental health problems based on early-life adversity (n=7,608) | | | | | | | | |
| --- | --- | --- | --- | --- | --- | --- | --- | --- |
| **Mental Health Outcome (Lifetime)** | **Weighted Prevalence** | **AUC**  **(Continuous Adversity Risk Calculation)** | **Dichotomous ELA Risk Classification** | | | | | |
|  |  |  | **ELA Cutoff** | **Odds Ratio (95% CI)** | **Sensitivity** | **Specificity** | **PPV** | **NPV** |
| **Anxiety** | 20.91 | 0.62 | ≥1 | 2.02 (1.74–2.34) | 0.62 (0.59–0.66) | 0.55 (0.53–0.56) | 0.27 (0.25–0.29) | 0.85 (0.83–0.86) |
|  |  |  | ≥2 | 2.52 (2.18–2.91) | 0.41 (0.38–0.44) | 0.78 (0.77–0.80) | 0.33 (0.31–0.36) | 0.83 (0.82–0.85) |
|  |  |  | ≥3 | 2.76 (2.34–3.26) | 0.27 (0.24–0.29) | 0.88 (0.87–0.89) | 0.38 (0.34–0.41) | 0.82 (0.81–0.83) |
|  |  |  | ≥4^‡^ | - | – | – | - | - |
| **Mood** | 14.68 | 0.64 | ≥1 | 2.25 (1.91–2.66) | 0.66 (0.62–0.69) | 0.54 (0.53–0.56) | 0.20 (0.18–0.21) | 0.90 (0.89–0.91) |
|  |  |  | ≥2 | 2.85 (2.42–3.36) | 0.45 (0.41–0.49) | 0.78 (0.76–0.79) | 0.26 (0.23–0.28) | 0.89 (0.88–0.90) |
|  |  |  | ≥3 | 3.08 (2.56–3.71) | 0.30 (0.26–0.33) | 0.88 (0.87–0.89) | 0.30 (0.26–0.33) | 0.88 (0.87–0.89) |
|  |  |  | ≥4 | 3.34 (2.59–4.31) | 0.16 (0.13–0.19) | 0.95 (0.94–0.95) | 0.34 (0.28–0.39) | 0.87 (0.86–0.88) |
| **SUD** | 20.16 | 0.67 | ≥1 | 2.69 (2.29–3.17) | 0.68 (0.65–0.71) | 0.56 (0.55–0.58) | 0.28 (0.26–0.30) | 0.87 (0.86–0.89) |
|  |  |  | ≥2 | 2.55 (2.17–2.99) | 0.41 (0.38–0.45) | 0.78 (0.77–0.80) | 0.33 (0.30–0.35) | 0.84 (0.83–0.85) |
|  |  |  | ≥3 | 2.35 (1.95–2.84) | 0.25 (0.22–0.28) | 0.88 (0.87–0.89) | 0.34 (0.30–0.37) | 0.82 (0.81–0.83) |
|  |  |  | ≥4 | 2.22 (1.73–2.86) | 0.12 (0.10–0.14) | 0.94 (0.93–0.95) | 0.34 (0.29–0.40) | 0.81 (0.80–0.82) |
| **Suicidality** | 10.53 | 0.63 | ≥1 | 3.19 (2.58–3.95) | 0.73 (0.69–0.77) | 0.54 (0.53–0.56) | 0.16 (0.14–0.17) | 0.94 (0.94–0.95) |
|  |  |  | ≥2 | 3.22 (2.67–3.88) | 0.49 (0.45–0.53) | 0.77 (0.76–0.78) | 0.20 (0.18–0.22) | 0.93 (0.92–0.94) |
|  |  |  | ≥3 | 3.67 (3.00–4.51) | 0.34 (0.30–0.38) | 0.88 (0.87–0.89) | 0.25 (0.21–0.28) | 0.92 (0.91–0.93) |
|  |  |  | ≥4 | 3.27 (2.50–4.29) | 0.17 (0.14–0.20) | 0.94 (0.93–0.95) | 0.25 (0.21–0.30) | 0.91 (0.90–0.91) |
| *^‡^Ineligible for release from Statistics Canada dataset due to vetting sample size rules*  ELA: Early-life Adversity  CI: Confidence interval  NPV: Negative predictive value  PPV: Positive predictive value  SUD: Substance use disorder | | | | | | | | |


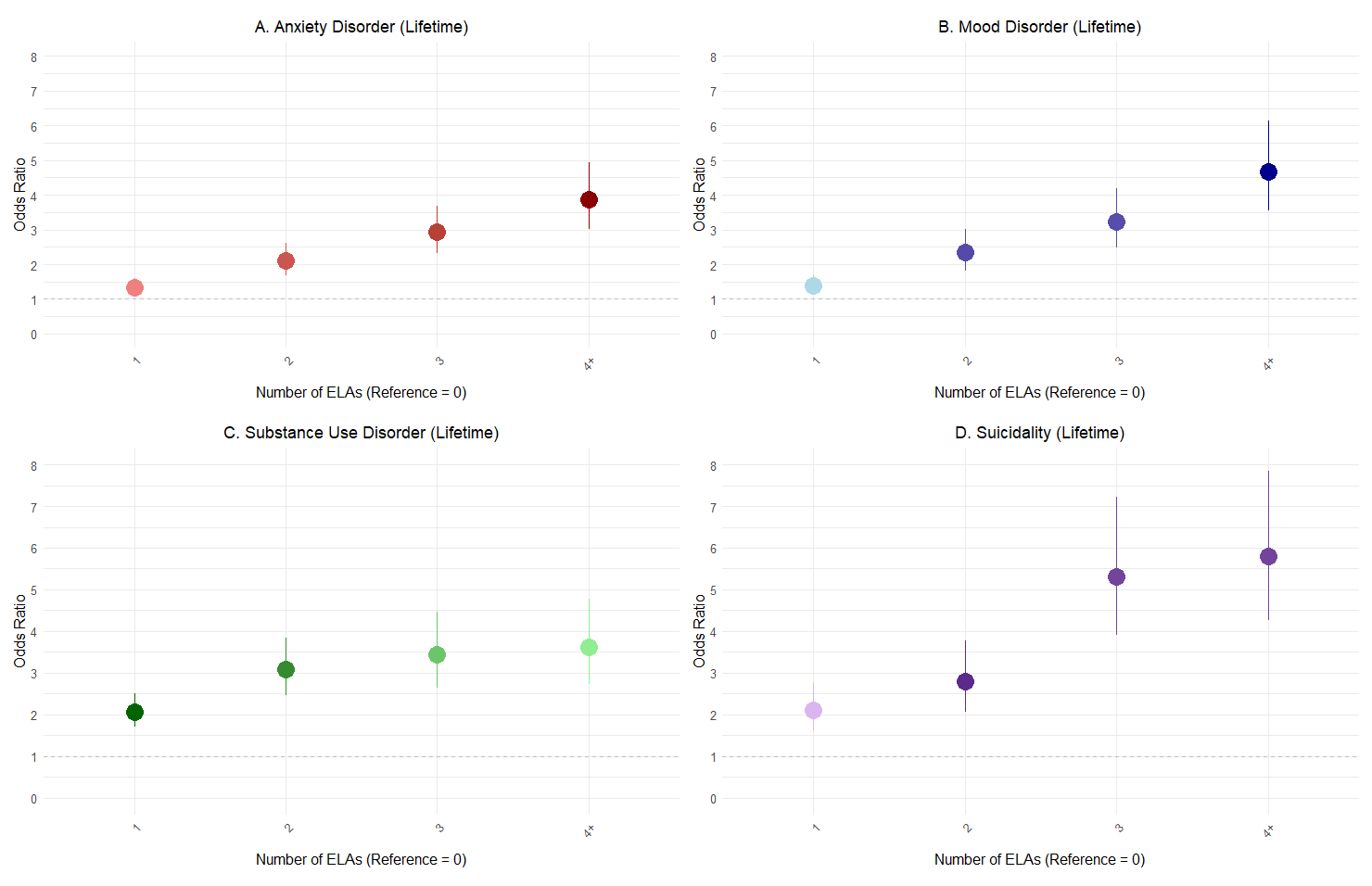
***Figure S1.*** Bivariate logistic regression model results on the association between number of ELAs and lifetime mental health problems (n=7,608). ELA prevalences were: 0 (51.2%), 1 (23.1%), 2 (11.0%), 3 (7.7%), ≥4 (7.0%).

***
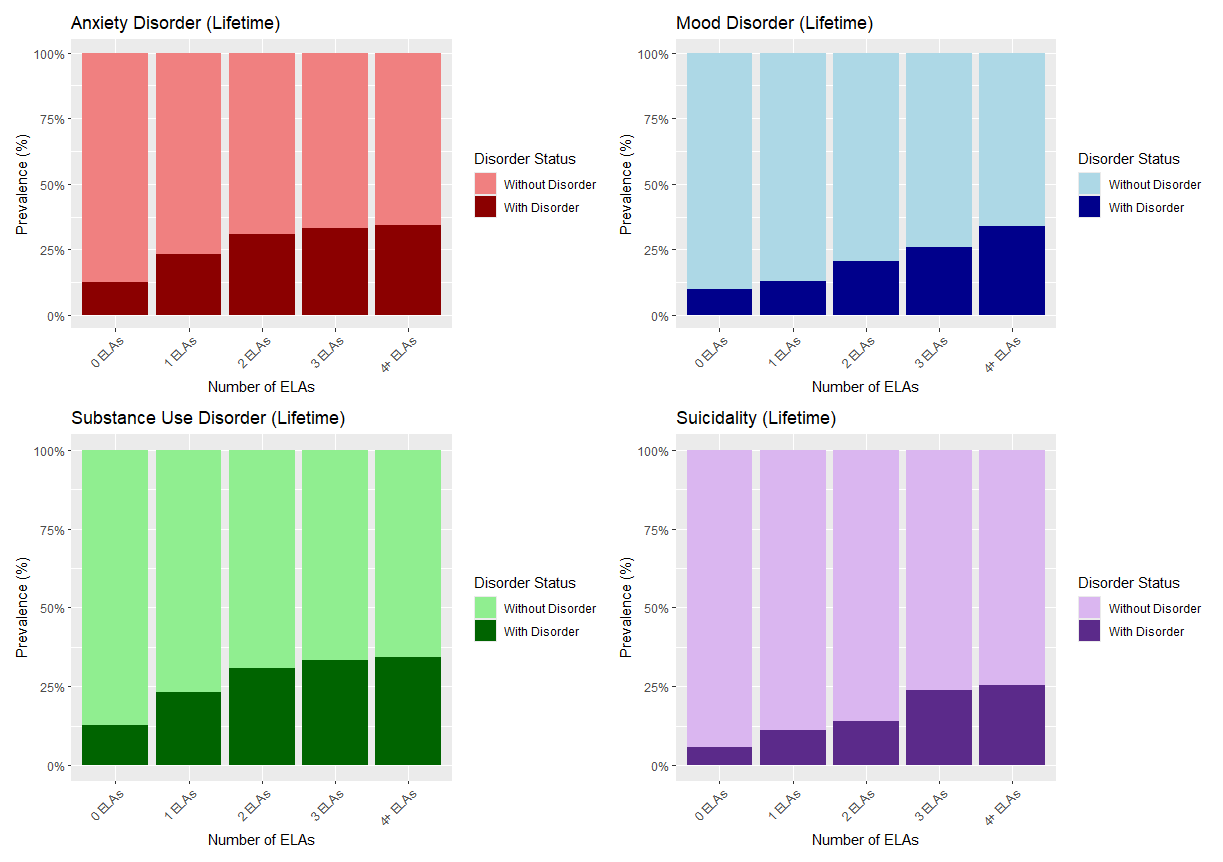
Figure S2****.* Prevalence of lifetime mental health problems across levels of ELA. ELA prevalences were: 0 (51.2%), 1 (23.1%), 2 (11.0%), 3 (7.7%), ≥4 (7.0%).

Area Under the Curve (AUC) Models for Continuous ELAs Measure

Anxiety Disorder (Lifetime)


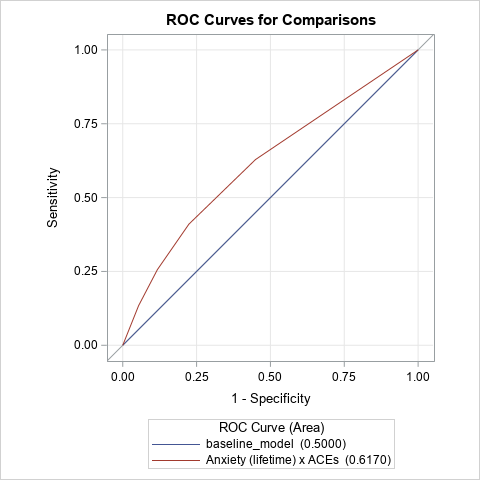


| **ROC Association Statistics** | | | | | | | |
| --- | --- | --- | --- | --- | --- | --- | --- |
| **ROC Model** | **Mann-Whitney** | | | | **Somers' D** | **Gamma** | **Tau-a** |
|  | **Area** | **Standard Error** | **95% Wald Confidence Limits** | |  |  |  |
| **baseline_model** | 0.5000 | 0 | 0.5000 | 0.5000 | 0 | . | 0 |
| **Anxiety (lifetime) x ELAs** | 0.6170 | 0.00818 | 0.6010 | 0.6331 | 0.2341 | 0.3274 | 0.0692 |

Depressive Disorder (Lifetime)


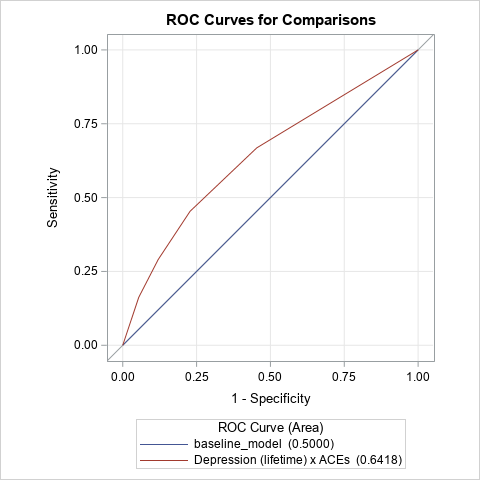


| **ROC Association Statistics** | | | | | | | |
| --- | --- | --- | --- | --- | --- | --- | --- |
| **ROC Model** | **Mann-Whitney** | | | | **Somers' D** | **Gamma** | **Tau-a** |
|  | **Area** | **Standard Error** | **95% Wald Confidence Limits** | |  |  |  |
| **baseline_model** | 0.5000 | 0 | 0.5000 | 0.5000 | 0 | . | 0 |
| **Depression (lifetime) x ELAs** | 0.6418 | 0.00935 | 0.6235 | 0.6601 | 0.2836 | 0.3856 | 0.0643 |

Suicidality (Lifetime)


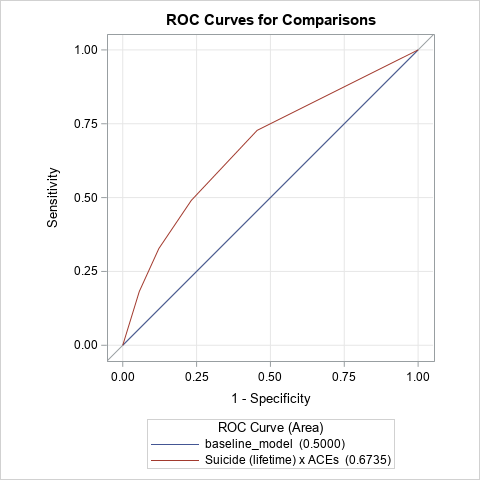


| **ROC Association Statistics** | | | | | | | |
| --- | --- | --- | --- | --- | --- | --- | --- |
| **ROC Model** | **Mann-Whitney** | | | | **Somers' D** | **Gamma** | **Tau-a** |
|  | **Area** | **Standard Error** | **95% Wald Confidence Limits** | |  |  |  |
| **baseline_model** | 0.5000 | 0 | 0.5000 | 0.5000 | 0 | . | 0 |
| **Suicide (lifetime) x ELAs** | 0.6735 | 0.0103 | 0.6533 | 0.6936 | 0.3469 | 0.4559 | 0.0611 |

Substance Use Disorder (Lifetime)


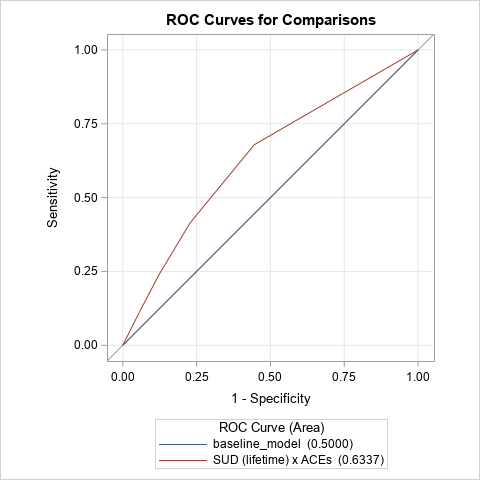


| **ROC Association Statistics** | | | | | | | |
| --- | --- | --- | --- | --- | --- | --- | --- |
| **ROC Model** | **Mann-Whitney** | | | | **Somers' D** | **Gamma** | **Tau-a** |
|  | **Area** | **Standard Error** | **95% Wald Confidence Limits** | |  |  |  |
| **baseline_model** | 0.5000 | 0 | 0.5000 | 0.5000 | 0 | . | 0 |
| **SUD (lifetime) x ELAs** | 0.6337 | 0.00836 | 0.6173 | 0.6500 | 0.2673 | 0.3654 | 0.0701 |

Anxiety Disorder (Past 12 Months)


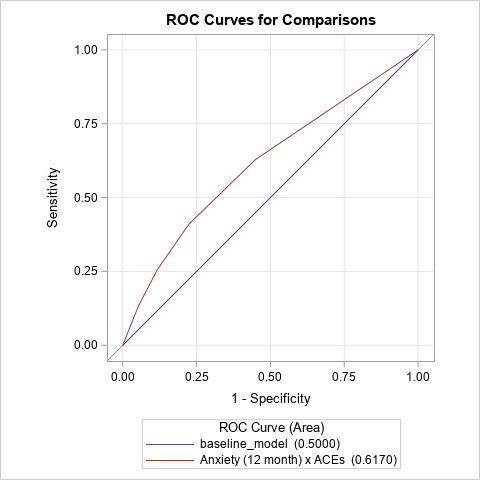


| **ROC Association Statistics** | | | | | | | |
| --- | --- | --- | --- | --- | --- | --- | --- |
| **ROC Model** | **Mann-Whitney** | | | | **Somers' D** | **Gamma** | **Tau-a** |
|  | **Area** | **Standard Error** | **95% Wald Confidence Limits** | |  |  |  |
| **baseline_model** | 0.5000 | 0 | 0.5000 | 0.5000 | 0 | . | 0 |
| **Anxiety (12 month) x ELAs** | 0.6170 | 0.00818 | 0.6010 | 0.6331 | 0.2341 | 0.3274 | 0.0692 |

Depressive Disorder (Past 12 Months)


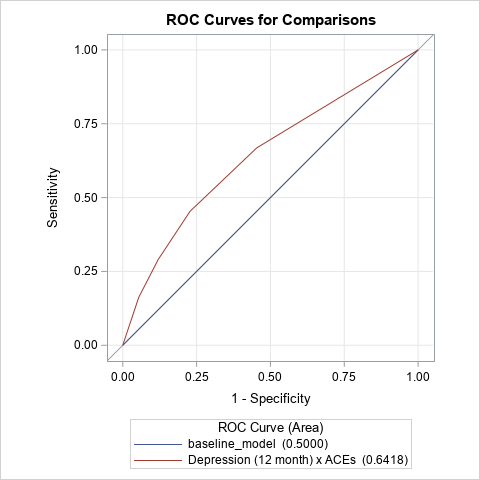


| **ROC Association Statistics** | | | | | | | |
| --- | --- | --- | --- | --- | --- | --- | --- |
| **ROC Model** | **Mann-Whitney** | | | | **Somers' D** | **Gamma** | **Tau-a** |
|  | **Area** | **Standard Error** | **95% Wald Confidence Limits** | |  |  |  |
| **baseline_model** | 0.5000 | 0 | 0.5000 | 0.5000 | 0 | . | 0 |
| **Depression (12 month) x ELAs** | 0.6418 | 0.00935 | 0.6235 | 0.6601 | 0.2836 | 0.3856 | 0.0643 |

Suicidality (Past 12 Months)


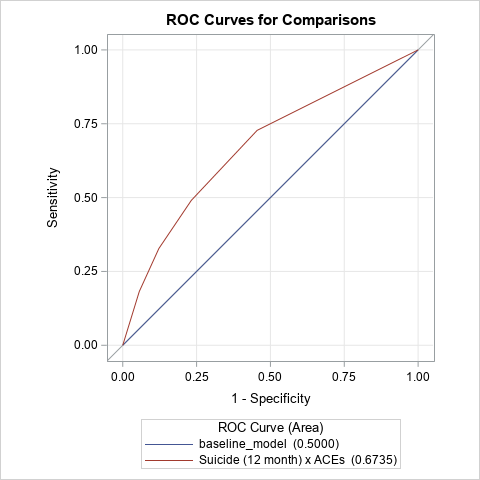


| **ROC Association Statistics** | | | | | | | |
| --- | --- | --- | --- | --- | --- | --- | --- |
| **ROC Model** | **Mann-Whitney** | | | | **Somers' D** | **Gamma** | **Tau-a** |
|  | **Area** | **Standard Error** | **95% Wald Confidence Limits** | |  |  |  |
| **baseline_model** | 0.5000 | 0 | 0.5000 | 0.5000 | 0 | . | 0 |
| **Suicide (12 month) x ELAs** | 0.6735 | 0.0103 | 0.6533 | 0.6936 | 0.3469 | 0.4559 | 0.0611 |

Substance Use Disorder (Past 12 Months)


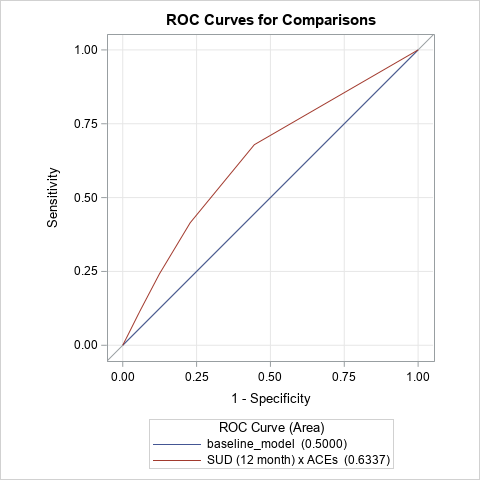


| **ROC Association Statistics** | | | | | | | |
| --- | --- | --- | --- | --- | --- | --- | --- |
| **ROC Model** | **Mann-Whitney** | | | | **Somers' D** | **Gamma** | **Tau-a** |
|  | **Area** | **Standard Error** | **95% Wald Confidence Limits** | |  |  |  |
| **baseline_model** | 0.5000 | 0 | 0.5000 | 0.5000 | 0 | . | 0 |
| **SUD (12 month) x ELAs** | 0.6337 | 0.00836 | 0.6173 | 0.6500 | 0.2673 | 0.3654 | 0.0701 |

**Sensitivity Analyses with *Any* Lifetime and Past 12-Month Psychiatric Outcome**

Here, we replicated both the past 12-month and lifetime analyses using *any psychiatric condition* as the outcome. The weighted prevalence of any *lifetime* psychiatric disorder was 39.59%, compared with 17.08% for any *past 12-month* psychiatric disorder (Tables S2–S3).

Consistent with the primary analyses, there was a dose–response relation between increasing ELA exposure and the likelihood of any psychiatric disorder for both lifetime and past 12-month outcomes. Odds of having any psychiatric disorder were elevated at all ELA cutoffs (≥1, ≥2, ≥3, ≥4) relative to those with no ELAs. The magnitude of association was somewhat larger for lifetime outcomes (e.g., OR=2.61 at ≥1 ELA; OR=3.05 at ≥4 ELAs) compared to past 12-month outcomes (OR=2.15 at ≥1 ELA; OR=2.85 at ≥4 ELAs), but was roughly comparable across timeframes.

In terms of individual risk prediction, discrimination based on the continuous adversity score was poor for both lifetime and past 12-month outcomes. For *lifetime* psychiatric disorder, the AUC was 0.64, indicating poor discrimination between those with and without a disorder. Similarly, discrimination for *past 12-month* psychiatric disorder was poor (AUC = 0.63). In both cases, predictive performance was only marginally better than chance, indicating that ELA exposure alone has limited utility for accurately distinguishing individuals with versus without psychiatric disorders at the individual level.

For classification accuracy using dichotomous ELA cutoffs, patterns of sensitivity, specificity, PPV, and NPV were broadly similar across lifetime and past 12-month outcomes, with expected differences reflecting base rate prevalence. Focusing on the high-risk cutoff of ≥4 ELAs, sensitivity was very low for both lifetime (0.12) and past 12-month (0.14) outcomes, indicating that this threshold failed to identify most individuals with a psychiatric disorder (i.e., low true positive rate). In contrast, specificity was high for both lifetime (0.96) and past 12-month (0.95) outcomes, indicating that having fewer than four ELAs accurately identified most individuals without a disorder. PPV values were higher for lifetime outcomes (range across cutoffs = 0.51–0.65) than for past 12-month outcomes (0.23–0.35), reflecting the much higher base rate of lifetime psychiatric disorders. Nonetheless, even for lifetime outcomes, a substantial proportion of individuals with ≥4 ELAs did not have a psychiatric disorder, indicating that high ELA exposure does not imply high probability of disorder at the individual level. Conversely, NPV values were relatively high for both lifetime (0.62–0.71) and past 12-month (0.84–0.88) outcomes, though NPVs were lower for lifetime outcomes due to their higher overall prevalence.

These results are largely consistent with those presented in the main text in suggesting clear evidence of increasing mental health risk as a function of increasing levels of adversity at the population level, but poor individual risk prediction.

| ***Table S4*.** Predictive accuracy for lifetime mental health problems based on early-life adversity (n=7,608) | | | | | | | | |
| --- | --- | --- | --- | --- | --- | --- | --- | --- |
| **Mental Health Outcome (Lifetime)** | **Weighted Prevalence** | **AUC**  **(Continuous Adversity Risk Calculation)** | **Dichotomous ELA Risk Classification** | | | | | |
|  |  |  | **ELA Cutoff** | **Odds Ratio (95% CI)** | **Sensitivity** | **Specificity** | **PPV** | **NPV** |
| **Any Psychiatric Outcome** | 39.59 | 0.64 | ≥1 | 2.61 (2.32-2.94) | 0.63 (0.61-0.65) | 0.61 (0.59-0.62) | 0.51 (0.49-0.53) | 0.71 (0.70-0.73) |
|  |  |  | ≥2 | 3.09 (2.70-3.55) | 0.39 (0.37-0.41) | 0.83 (0.82-0.84) | 0.60 (0.57-0.63) | 0.67 (0.66-0.69) |
|  |  |  | ≥3 | 3.16 (2.68-3.72) | 0.24 (0.22-0.26) | 0.91 (0.90-0.92) | 0.64 (0.60-0.67) | 0.65 (0.63-0.66) |
|  |  |  | ≥4 | 3.05 (2.41-3.86) | 0.12 (0.10-0.13) | 0.96 (0.95-0.97) | 0.65 (0.60-0.70) | 0.62 (0.61-0.64) |
| ELA: Early-life adversity  CI: Confidence interval  NPV: Negative predictive value  PPV: Positive predictive value  SUD: Substance use disorder | | | | | | | | |

Any Psychiatric Outcome (Lifetime)


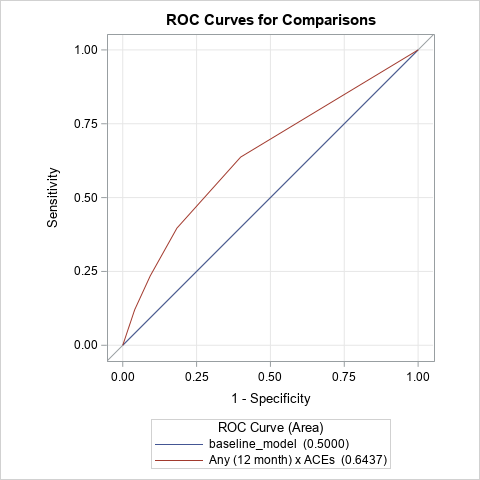


| **ROC Association Statistics** | | | | | | | |
| --- | --- | --- | --- | --- | --- | --- | --- |
| **ROC Model** | **Mann-Whitney** | | | | **Somers' D** | **Gamma** | **Tau-a** |
|  | **Area** | **Standard Error** | **95% Wald Confidence Limits** | |  |  |  |
| **baseline_model** | 0.5000 | 0 | 0.5000 | 0.5000 | 0 | . | 0 |
| **Any (12 month) x ELAs** | 0.6437 | 0.00630 | 0.6313 | 0.6560 | 0.2873 | 0.4078 | 0.1302 |

| ***Table S5.*** Predictive accuracy for past 12-month mental health problems based on early-life adversity (n=7,608) | | | | | | | | |
| --- | --- | --- | --- | --- | --- | --- | --- | --- |
| **Mental Health Outcome (Past 12-months)** | **Weighted Prevalence** | **AUC**  **(Continuous Adversity Risk Calculation)** | **Dichotomous Adversity Risk Classification** | | | | | |
|  |  |  | **ELA Cutoff** | **Odds Ratio (95% CI)** | **Sensitivity** | **Specificity** | **PPV** | **NPV** |
| **Any Psychiatric Outcome** | 17.08 | 0.63 | ≥1 | 2.15 (1.84-2.52) | 0.64 (0.61-0.68) | 0.54 (0.53-0.56) | 0.23 (0.21-0.24) | 0.88 (0.87-0.89) |
|  |  |  | ≥2 | 2.56 (2.18-2.99) | 0.42 (0.39-0.46) | 0.78 (0.77-0.79) | 0.28 (0.26-0.31) | 0.87 (0.86-0.88) |
|  |  |  | ≥3 | 2.55 (2.12-3.08) | 0.26 (0.87-0.89) | 0.88 (0.87-0.89) | 0.31 (0.27-0.34) | 0.85 (0.84-0.86) |
|  |  |  | ≥4 | 2.85 (2.22-3.66) | 0.14 (0.12-0.17) | 0.95 (0.94-0.95) | 0.35 (0.29-0.40) | 0.84 (0.83-0.85) |
| ELA: Early-life adversity  CI: Confidence interval  NPV: Negative predictive value  PPV: Positive predictive value  SUD: Substance use disorder | | | | | | | | |

Any Psychiatric Outcome (Past 12 Months)


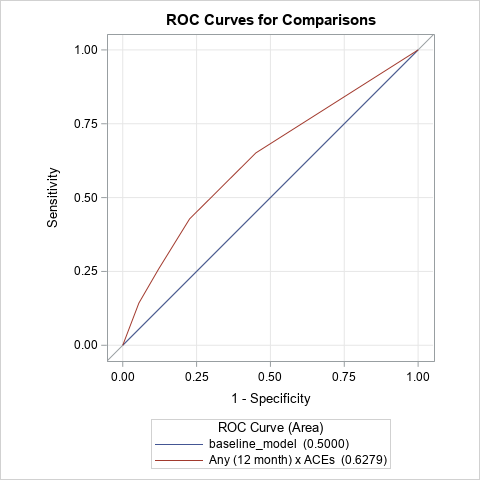


| **ROC Association Statistics** | | | | | | | |
| --- | --- | --- | --- | --- | --- | --- | --- |
| **ROC Model** | **Mann-Whitney** | | | | **Somers' D** | **Gamma** | **Tau-a** |
|  | **Area** | **Standard Error** | **95% Wald Confidence Limits** | |  |  |  |
| **baseline_model** | 0.5000 | 0 | 0.5000 | 0.5000 | 0 | . | 0 |
| **Any (12 month) x ELAs** | 0.6279 | 0.00865 | 0.6110 | 0.6448 | 0.2558 | 0.3528 | 0.0669 |
